# The impact of imprecisely measured covariates on estimating gene-environment interactions

**DOI:** 10.1186/1471-2288-6-21

**Published:** 2006-05-04

**Authors:** Darren C Greenwood, Mark S Gilthorpe, Janet E Cade

**Affiliations:** 1 Biostatistics Unit, University of Leeds, Leeds, UK; 2 Nutrition Epidemiology Group, University of Leeds, Leeds, UK

## Abstract

**Background:**

The effects of measurement error in epidemiological exposures and confounders on estimated effects of exposure are well described, but the effects on estimates for gene-environment interactions has received rather less attention. In particular, the effects of confounder measurement error on gene-environment interactions are unknown.

**Methods:**

We investigate these effects using simulated data and illustrate our results with a practical example in nutrition epidemiology.

**Results:**

We show that the interaction regression coefficient is unchanged by confounder measurement error under certain conditions, but biased by exposure measurement error. We also confirm that confounder measurement error can lead to estimated effects of exposure biased either towards or away from the null, depending on the correlation structure, with associated effects on type II errors.

**Conclusion:**

Whilst measurement error in confounders does not lead to bias in interaction coefficients, it may still lead to bias in the estimated effects of exposure. There may still be cost implications for epidemiological studies that need to calibrate all error-prone covariates against a valid reference, in addition to the exposure, to reduce the effects of confounder measurement error.

## Background

One of the largest difficulties facing epidemiological research is that of measurement error in an exposure or relevant confounders [1-4]. Measurement error can lead to substantial bias in either direction, either diluting or exaggerating the apparent effect size [5]. There is a particular problem in the area of nutrition epidemiology where measuring long-term dietary intake is prone to error, such that most epidemiological studies in this field are subject potentially to very large biases [6,7]. An additional side-effect of measurement error is reduction in statistical power – the ability to detect a true difference of practical importance [8-11]. Whilst these effects of measurement error in exposures are well described, the effects of measurement error in confounding variables have received less attention [5,12-16].

The source of measurement error may occur in the assessment tool used to determine the extent of exposure or dietary confounder. For example, food frequency questionnaires may use crude measures of portion size, frequency of consumption, and use broad food groupings, which all limit the precision with which dietary intake can be estimated. In addition, the source of error could be random variation in the exposure attributable to chance fluctuations, and not dependent on the assessment tool. In this way natural variation in individuals' diets from day-to-day and week-to-week could lead to random error in estimating long-term dietary intake. For example, a food diary or a series of 24 hour recalls may record actual intake more precisely than a food frequency questionnaire (FFQ), but only represents a short period of time so will lack precision compared to true long-term intake. Another source of error could be related to the individual completing the dietary assessment, leading to a person-specific bias and measurement errors in two instruments being correlated [17-21].

One area of epidemiology receiving increasing attention is that of the gene-environment interaction. The researcher is often interested in whether an epidemiological exposure has a different effect dependent on an individual's genotype. Alternatively, they may want to identify groups, identifiable on the basis of genotype or phenotype, at greater risk from a particular exposure. One type of gene-environment interaction that can be investigated is the gene-diet interaction, where the environmental exposure is a particular dietary intake. Whilst the effects of measurement error on estimation procedures such as linear regression are well known for main effects, the influence of errors on estimation of interaction terms is not well documented. In particular, the effect of measurement error in confounding variables on a statistical interaction is unknown.

We aim to characterise the impact of measurement error in an exposure and in a confounder in the estimation of both main effects as well as their interaction. We present a series of simulations demonstrating the effect of measurement error in a variety of situations. We illustrate our findings with a recent cohort study where we investigate the relationship between HFE genotype for haemochromatosis (iron overload), diet, and serum ferritin concentrations [22].

## Methods

### Simulations

We denote the true covariate, *X*, and its surrogate, *W*, measured with error *U *under the classical additive measurement error model such that *W *= *X *+ *U*. We assume *X*~N(0,1), *U*~N(0, *σ*_u_^2^), and that given *X*, *W *contributes no additional information about the outcome, *Y*. This means that, in terms of conditional probability distributions, *f*(Y|X, W) = *f*(Y|X). In addition we represent the genotype, *G*, as coded 1 for homozygotes and 0 for heterozygotes and wild types, where *G*~bernoulli(p). We assume p = 0.2. We generate a potential confounding variable, *C*, such that C~N(0,1), corr(X, C) = *ρ*_xc_, corr(Y, C) = *ρ*_yc_, and *C*'s surrogate, *D*, is measured with error such that *D *= *C *+ *V*, where measurement error *V*~N(0, *σ*_v_^2^). For each scenario, we generate n observations such that *Y *= *β*_0 _+ *β*_1_*G *+ *β*_2_*X *+ *β*_3 _*G.X *+ *β*_4 _*C *+ *ε*, where *ε *represents residual error. For the purposes of estimating standard deviations of estimates and the probability of rejecting the null hypothesis H_0_, we assume the residual error *ε*~N(0,4). Parameters were chosen to give reasonable R^2 ^values approximately in the range 10–25%, based on experience in the UK Women's Cohort [23], and dependent on the scenario and amount of measurement error in the exposure and confounder. To achieve adequate precision in estimates, 10000 simulations were performed for each scenario, with each containing a sample size of 1000 observations. The regression model intercept is set to *β*_0 _= 0 for all simulations. An interaction can be expressed in terms of either the regression coefficient *β*_3 _above, referred to here as the coefficient estimate, or alternatively as the ratio of the regression slopes for each genotype, where the ratio = (*β*_2 _+ *β*_3_)/*β*_2 _is referred to here as the ratio estimate.

For the simulations, measurement error magnitude can be expressed in four different ways: (i) as the measurement error variance (e.g. *σ*_u_^2^), (ii) as the reliability ratio, *λ *(e.g. *σ*_x_^2^/(*σ*_x_^2 ^+ *σ*_u_^2^)), (iii) as the correlation between repeated measures of the covariate, which is mathematically equivalent to *λ *[24], and (iv) as the correlation between the imperfectly measured covariate and its true values, mathematically equivalent to √*λ *[24].

#### Scenario 1

The initial aim is to investigate the effect of measurement error in a confounding variable on the coefficient of a perfectly measured exposure and on the interaction between a perfectly measured exposure and a perfectly measured genotype. For scenario 1 we assume that *X *is measured without error, i.e. *U *= 0, hence *W *= *X*, and that the true effect of exposure *X *is such that *β*_2 _= 1. We also assume the true genotype effect to be *β*_1 _= 1 and the true interaction between *X *and *G *is such that *β*_3 _= 1. The effect of the confounder, *β*_4_, is set to either 1 or -1. For scenario 1 the data were therefore generated from *Y *= *β*_0 _+ *β*_1_*G *+ *β*_2_*X *+ *β*_3_*G.X *+ *β*_4 _*C *+ *ε *and the regression model fit to these data was the same but with *C *replaced by its surrogate *D*. We consider correlations between confounder *C *and exposure *X *of 0.2, 0.5 and -0.5. The measurement error variance, *σ*_v_^2^, in the confounding variable *D *was set to 0, 1, 2, 4, and 9 (equivalent to reliability ratios, *λ*, of 1.0, 0.5, 0.33, 0.2 and 0.1 respectively). An alternative way of viewing the latter is to consider a replicate measurement on the same individuals, *D'*, subject to the same level of measurement error. The correlation between *D *and *D' *would have the same values as the reliability ratios, that is 1.0, 0.5, 0.33, 0.2 and 0.1 respectively. Alternatively, this could also be presented in terms of the correlation between the true and observed confounders *C *and *D*, equivalent to √ *λ*, having values 1.0, 0.71, 0.58, 0.45 and 0.32.

#### Scenario 2

The second aim was to investigate the effect of measurement error in an exposure on estimates of the interaction between the exposure and a perfectly measured genotype. We assume that *X *is now measured with error, hence *U*~N(0, *σ*_u_^2^), and *W *= *X+U*, with the true effect of exposure *X *such that *β*_2 _= 1. We assume the true effect of genotype to be *β*_1 _= 1. Estimates of model coefficients and the probability of rejecting H_0 _are investigated for true interactions between *X *and *G *of *β*_3 _= 0, 0.5, 1 and 2 (equivalent to ratios of the two regression slopes of 1, 1.5, 2, and 3 respectively). In this scenario we assume that the exposure is not subject to confounding, i.e. *β*_4 _= 0. For scenario 2 the data were therefore generated from *Y *= *β*_0 _+ *β*_1_*G *+ *β*_2_*X *+ *β*_3 _*G.X *+ *ε *and the regression model fit to these data was the same but with *X *replaced by its surrogate *W*.

### Practical illustration

Detailed methods have been presented elsewhere [22] and are briefly summarised here. We sought to determine the relationship between haem iron intake (from meat), iron storage status and the risk of iron accumulation in subjects who are carriers of certain genetic mutations associated with haemochromatosis, a hereditary condition characterised by excessively high iron stores potentially leading to severe chronic diseases. For this illustration we focus on mutations of the C282Y genotype, combining heterozygotes and wild types into one category, and comparing with homozygotes. We assume the assessment of genotype is perfectly measured and that combining heterozygotes and wild types does not introduce any measurement error. Participants were sampled from the UK Women's Cohort Study, a cohort of 35 372 women living in the United Kingdom aged 35–69 in 1995 [23]. Blood samples were available for 2489 women, giving serum ferritin concentrations and C282Y genotype. Intake of haem iron and other nutrients were measured using a 217 item FFQ [23,25,26]. A second FFQ was completed by 820 (33%) of these approximately 5 years after the first. This gap minimises correlation between the measurement errors in each response. The drift over time in response between the two measures was taken into account by subtracting the difference between the mean responses from the second FFQ results as suggested by Carroll et al[3] and Landin et al[27].

Linear regression was used to explore the relationship between log-transformed serum ferritin concentrations (as a measure of iron storage) and haem iron intake. Several potential confounders were identified [22]. However, for the purpose of illustration, only the main one, total energy intake, is included in the model, along with the two main effects (genotype and haem iron intake), and their interaction. In the presence of the interaction term, the main effect of the exposure is interpreted as the exposure effect in the genotype referent group. The influence of genotype on the relationship between haem iron intake and serum ferritin was formally tested by adding their interaction to the model.

Measurement error was adjusted for by regression calibration [28,29] using Stata version 8 [30]. However, the preponderance of zeros in the interaction component introduced by multiplying the dummy variable for the perfectly measured genotype by the continuous exposure can lead to model instability (data not shown). In terms of regression calibration it is more robust to treat the interaction component, not as a separate error-prone variable forming a second variable to include in the regression calibration, but to base it on E(*X*|*W*,*G*) derived for the exposure variable. This approach provides a function that meets the requirements for regression calibration, yielding more robust results (data not shown).

## Results

### Scenario 1

When the exposure and error-prone confounder are positively correlated (i.e. *ρ*_xc_>0) and both act in same direction on the outcome (i.e. *β*_4 _has the same sign as *β*_2_), or if they are negatively correlated (i.e. *ρ*_xc_<0) and acting in opposite directions (i.e. *β*_4 _has opposite sign to *β*_2_), then the measurement error in the confounder causes the estimated effect of exposure to be biased away from the null, even when the exposure is measured without error (Table 1). For moderately sized correlations, the bias can be substantial. Though estimated coefficients vary greatly, the empirical standard deviation of the estimates remained similar, approximately 0.07 or 0.08, with the smallest for the situation with no measurement error in the confounder and weak correlation between the true values of the confounder and exposure. Therefore, unlike measurement error in an exposure, which leads to lower probability of rejecting H_0 _[8-11], measurement error in a confounder can lead to either increased or decreased probability of rejecting H_0 _when assessing the effect of the exposure [31,32]. It achieves this by biasing estimates either away from (increased probability of rejecting H_0_) or towards (decreased probability of rejecting H_0_) the null, depending on the effects of the residual confounding [31,33-35]. However, any increased probability of rejecting H_0 _is due to bias, and does not reflect true statistical power. The effect of measurement error in the confounder on the estimated exposure effect β^
 MathType@MTEF@5@5@+=feaafiart1ev1aaatCvAUfKttLearuWrP9MDH5MBPbIqV92AaeXatLxBI9gBaebbnrfifHhDYfgasaacH8akY=wiFfYdH8Gipec8Eeeu0xXdbba9frFj0=OqFfea0dXdd9vqai=hGuQ8kuc9pgc9s8qqaq=dirpe0xb9q8qiLsFr0=vr0=vr0dc8meaabaqaciaacaGaaeqabaqabeGadaaakeaaiiGacuWFYoGygaqcaaaa@2E64@_2 _was unaltered by excluding genotype or the interaction term from the model. The estimated coefficient associated with the confounder, β^
 MathType@MTEF@5@5@+=feaafiart1ev1aaatCvAUfKttLearuWrP9MDH5MBPbIqV92AaeXatLxBI9gBaebbnrfifHhDYfgasaacH8akY=wiFfYdH8Gipec8Eeeu0xXdbba9frFj0=OqFfea0dXdd9vqai=hGuQ8kuc9pgc9s8qqaq=dirpe0xb9q8qiLsFr0=vr0=vr0dc8meaabaqaciaacaGaaeqabaqabeGadaaakeaaiiGacuWFYoGygaqcaaaa@2E64@_4_, was subject to the usual bias caused by measurement error. The estimated effects of genotype β^
 MathType@MTEF@5@5@+=feaafiart1ev1aaatCvAUfKttLearuWrP9MDH5MBPbIqV92AaeXatLxBI9gBaebbnrfifHhDYfgasaacH8akY=wiFfYdH8Gipec8Eeeu0xXdbba9frFj0=OqFfea0dXdd9vqai=hGuQ8kuc9pgc9s8qqaq=dirpe0xb9q8qiLsFr0=vr0=vr0dc8meaabaqaciaacaGaaeqabaqabeGadaaakeaaiiGacuWFYoGygaqcaaaa@2E64@_1 _and the coefficient estimate of interaction β^
 MathType@MTEF@5@5@+=feaafiart1ev1aaatCvAUfKttLearuWrP9MDH5MBPbIqV92AaeXatLxBI9gBaebbnrfifHhDYfgasaacH8akY=wiFfYdH8Gipec8Eeeu0xXdbba9frFj0=OqFfea0dXdd9vqai=hGuQ8kuc9pgc9s8qqaq=dirpe0xb9q8qiLsFr0=vr0=vr0dc8meaabaqaciaacaGaaeqabaqabeGadaaakeaaiiGacuWFYoGygaqcaaaa@2E64@_3 _were themselves unaffected by confounder measurement error. This is because the confounder is uncorrelated with genotype and, conditional on the other terms in the model, the confounder is uncorrelated with the interaction. This means that the confounder for the exposure is not a confounder for genotype or for the interaction, so adjustment for the confounder is unnecessary for unbiased estimation of their coefficients. However, because β^
 MathType@MTEF@5@5@+=feaafiart1ev1aaatCvAUfKttLearuWrP9MDH5MBPbIqV92AaeXatLxBI9gBaebbnrfifHhDYfgasaacH8akY=wiFfYdH8Gipec8Eeeu0xXdbba9frFj0=OqFfea0dXdd9vqai=hGuQ8kuc9pgc9s8qqaq=dirpe0xb9q8qiLsFr0=vr0=vr0dc8meaabaqaciaacaGaaeqabaqabeGadaaakeaaiiGacuWFYoGygaqcaaaa@2E64@_2 _varied whilst β^
 MathType@MTEF@5@5@+=feaafiart1ev1aaatCvAUfKttLearuWrP9MDH5MBPbIqV92AaeXatLxBI9gBaebbnrfifHhDYfgasaacH8akY=wiFfYdH8Gipec8Eeeu0xXdbba9frFj0=OqFfea0dXdd9vqai=hGuQ8kuc9pgc9s8qqaq=dirpe0xb9q8qiLsFr0=vr0=vr0dc8meaabaqaciaacaGaaeqabaqabeGadaaakeaaiiGacuWFYoGygaqcaaaa@2E64@_3 _remained constant, the ratio estimate of the interaction [(*β*_2 _+ *β*_3_)/*β*_2_] varied with confounder measurement error. Since measurement error in the confounder has no noticeable effect on either the estimate of the interaction coefficient or the empirical standard deviation of the estimates, the power for this assessment is unaffected. This also holds for the ratio estimate of interaction. Monte Carlo error was 1% of the empirical standard deviation of the estimates, giving adequate precision in the estimates to two decimal places.

**Table 1:** Scenario 1: The effect of measurement error *σ*_v_^2 ^in a confounding variable on estimated exposure effects β^
 MathType@MTEF@5@5@+=feaafiart1ev1aaatCvAUfKttLearuWrP9MDH5MBPbIqV92AaeXatLxBI9gBaebbnrfifHhDYfgasaacH8akY=wiFfYdH8Gipec8Eeeu0xXdbba9frFj0=OqFfea0dXdd9vqai=hGuQ8kuc9pgc9s8qqaq=dirpe0xb9q8qiLsFr0=vr0=vr0dc8meaabaqaciaacaGaaeqabaqabeGadaaakeaaiiGacuWFYoGygaqcaaaa@2E64@_2_. The exposure is measured without error.

Coefficient for true effect of confounder (*β*_4_)	Correlation between true value of confounder and exposure	β^ MathType@MTEF@5@5@+=feaafiart1ev1aaatCvAUfKttLearuWrP9MDH5MBPbIqV92AaeXatLxBI9gBaebbnrfifHhDYfgasaacH8akY=wiFfYdH8Gipec8Eeeu0xXdbba9frFj0=OqFfea0dXdd9vqai=hGuQ8kuc9pgc9s8qqaq=dirpe0xb9q8qiLsFr0=vr0=vr0dc8meaabaqaciaacaGaaeqabaqabeGadaaakeaaiiGacuWFYoGygaqcaaaa@2E64@_2 _(sd of estimate)				
		
		*σ*_v_^2 ^= 0	*σ*_v_^2 ^= 1	*σ*_v_^2 ^= 2	*σ*_v_^2 ^= 4	*σ*_v_^2 ^= 9
1.0	0.2	1.001 (0.073)	1.103 (0.076)	1.136 (0.077)	1.162 (0.078)	1.182 (0.079)
	0.5	1.001 (0.081)	1.287 (0.079)	1.365 (0.079)	1.422 (0.078)	1.463 (0.078)
	-0.5	1.000 (0.080)	0.715 (0.079)	0.637 (0.079)	0.580 (0.078)	0.539 (0.078)
-1.0	0.2	1.001 (0.073)	0.898 (0.077)	0.865 (0.078)	0.839 (0.078)	0.819 (0.079)
	0.5	1.001 (0.081)	0.715 (0.079)	0.637 (0.079)	0.579 (0.078)	0.539 (0.078)
	-0.5	1.000 (0.080)	1.286 (0.080)	1.364 (0.079)	1.421 (0.079)	1.462 (0.078)

True values of coefficients *β*_1 _= 1 (binary genotype), *β*_2 _= 1 (continuous exposure), *β*_3 _= 1 (interaction term), *β*_4 _(confounder) given in first column. Simulations based on 10,000 simulations of 1000 observations. Monte Carlo error is 1% of the empirical standard deviation of the estimates for β^
 MathType@MTEF@5@5@+=feaafiart1ev1aaatCvAUfKttLearuWrP9MDH5MBPbIqV92AaeXatLxBI9gBaebbnrfifHhDYfgasaacH8akY=wiFfYdH8Gipec8Eeeu0xXdbba9frFj0=OqFfea0dXdd9vqai=hGuQ8kuc9pgc9s8qqaq=dirpe0xb9q8qiLsFr0=vr0=vr0dc8meaabaqaciaacaGaaeqabaqabeGadaaakeaaiiGacuWFYoGygaqcaaaa@2E64@_2_; approximately 0.0008.

### Scenario 2

Measurement error in an exposure leads to bias in the coefficient estimate of interaction between that exposure and a perfectly measured genotype (Table 2). Where there is no confounding the interaction term tends to be under-estimated because on average it is biased towards the null, thereby diluting its apparent impact. The estimate of the exposure effect is under-estimated to the same degree, such that the ratio estimate of the interaction remains unaffected by measurement error in the exposure. Standard errors decrease, giving a false sense of precision. However, because the coefficient estimate is attenuated towards the null, the power is substantially decreased despite reduced standard errors (Table 3).

**Table 2:** The effect of measurement error in an exposure *σ*_u_^2 ^on estimated exposure (β^
 MathType@MTEF@5@5@+=feaafiart1ev1aaatCvAUfKttLearuWrP9MDH5MBPbIqV92AaeXatLxBI9gBaebbnrfifHhDYfgasaacH8akY=wiFfYdH8Gipec8Eeeu0xXdbba9frFj0=OqFfea0dXdd9vqai=hGuQ8kuc9pgc9s8qqaq=dirpe0xb9q8qiLsFr0=vr0=vr0dc8meaabaqaciaacaGaaeqabaqabeGadaaakeaaiiGacuWFYoGygaqcaaaa@2E64@_2_) and interaction between the exposure and a perfectly measured genotype (β^
 MathType@MTEF@5@5@+=feaafiart1ev1aaatCvAUfKttLearuWrP9MDH5MBPbIqV92AaeXatLxBI9gBaebbnrfifHhDYfgasaacH8akY=wiFfYdH8Gipec8Eeeu0xXdbba9frFj0=OqFfea0dXdd9vqai=hGuQ8kuc9pgc9s8qqaq=dirpe0xb9q8qiLsFr0=vr0=vr0dc8meaabaqaciaacaGaaeqabaqabeGadaaakeaaiiGacuWFYoGygaqcaaaa@2E64@_3_).

Coefficient for true effect of interaction(*β*_3_)	Ratio for true effect of interaction(*β*_3_+*β*_2_)/*β*_2_	β^ MathType@MTEF@5@5@+=feaafiart1ev1aaatCvAUfKttLearuWrP9MDH5MBPbIqV92AaeXatLxBI9gBaebbnrfifHhDYfgasaacH8akY=wiFfYdH8Gipec8Eeeu0xXdbba9frFj0=OqFfea0dXdd9vqai=hGuQ8kuc9pgc9s8qqaq=dirpe0xb9q8qiLsFr0=vr0=vr0dc8meaabaqaciaacaGaaeqabaqabeGadaaakeaaiiGacuWFYoGygaqcaaaa@2E64@_2_(sd of estimate)				β^ MathType@MTEF@5@5@+=feaafiart1ev1aaatCvAUfKttLearuWrP9MDH5MBPbIqV92AaeXatLxBI9gBaebbnrfifHhDYfgasaacH8akY=wiFfYdH8Gipec8Eeeu0xXdbba9frFj0=OqFfea0dXdd9vqai=hGuQ8kuc9pgc9s8qqaq=dirpe0xb9q8qiLsFr0=vr0=vr0dc8meaabaqaciaacaGaaeqabaqabeGadaaakeaaiiGacuWFYoGygaqcaaaa@2E64@_3_(sd of estimate)			
			
		*σ*_u_^2 ^= 1	*σ*_u_^2 ^= 2	*σ*_u_^2 ^= 4	*σ*_u_^2 ^= 9	*σ*_u_^2 ^= 1	*σ*_u_^2 ^= 2	*σ*_u_^2 ^= 4	*σ*_u_^2 ^= 9
0.0	1.0	0.50 (0.05)	0.33 (0.04)	0.20 (0.03)	0.10 (0.02)	0.00 (0.12)	0.00 (0.10)	0.00 (0.08)	0.00 (0.06)
0.5	1.5	0.50 (0.05)	0.33 (0.04)	0.20 (0.03)	0.10 (0.02)	0.25 (0.13)	0.17 (0.11)	0.10 (0.09)	0.05 (0.06)
1.0	2.0	0.50 (0.05)	0.33 (0.05)	0.20 (0.03)	0.10 (0.02)	0.50 (0.14)	0.33 (0.12)	0.20 (0.09)	0.10 (0.07)
2.0	3.0	0.50 (0.05)	0.33 (0.04)	0.20 (0.04)	0.10 (0.03)	1.00 (0.16)	0.67 (0.14)	0.40 (0.11)	0.20 (0.08)

True values of coefficients *β*_1 _= 1 (binary genotype), *β*_2 _= 1 (continuous exposure), *β*_3 _(interaction term) given in first column. No confounding present, *β*_4 _= 0. Simulations based on 10,000 simulations of 1000 observations. Monte Carlo error is 1% of the empirical standard deviation of the estimates for β^
 MathType@MTEF@5@5@+=feaafiart1ev1aaatCvAUfKttLearuWrP9MDH5MBPbIqV92AaeXatLxBI9gBaebbnrfifHhDYfgasaacH8akY=wiFfYdH8Gipec8Eeeu0xXdbba9frFj0=OqFfea0dXdd9vqai=hGuQ8kuc9pgc9s8qqaq=dirpe0xb9q8qiLsFr0=vr0=vr0dc8meaabaqaciaacaGaaeqabaqabeGadaaakeaaiiGacuWFYoGygaqcaaaa@2E64@_2 _or β^
 MathType@MTEF@5@5@+=feaafiart1ev1aaatCvAUfKttLearuWrP9MDH5MBPbIqV92AaeXatLxBI9gBaebbnrfifHhDYfgasaacH8akY=wiFfYdH8Gipec8Eeeu0xXdbba9frFj0=OqFfea0dXdd9vqai=hGuQ8kuc9pgc9s8qqaq=dirpe0xb9q8qiLsFr0=vr0=vr0dc8meaabaqaciaacaGaaeqabaqabeGadaaakeaaiiGacuWFYoGygaqcaaaa@2E64@_3_; approximately 0.0005 for β^
 MathType@MTEF@5@5@+=feaafiart1ev1aaatCvAUfKttLearuWrP9MDH5MBPbIqV92AaeXatLxBI9gBaebbnrfifHhDYfgasaacH8akY=wiFfYdH8Gipec8Eeeu0xXdbba9frFj0=OqFfea0dXdd9vqai=hGuQ8kuc9pgc9s8qqaq=dirpe0xb9q8qiLsFr0=vr0=vr0dc8meaabaqaciaacaGaaeqabaqabeGadaaakeaaiiGacuWFYoGygaqcaaaa@2E64@_2 _and 0.0015 for β^
 MathType@MTEF@5@5@+=feaafiart1ev1aaatCvAUfKttLearuWrP9MDH5MBPbIqV92AaeXatLxBI9gBaebbnrfifHhDYfgasaacH8akY=wiFfYdH8Gipec8Eeeu0xXdbba9frFj0=OqFfea0dXdd9vqai=hGuQ8kuc9pgc9s8qqaq=dirpe0xb9q8qiLsFr0=vr0=vr0dc8meaabaqaciaacaGaaeqabaqabeGadaaakeaaiiGacuWFYoGygaqcaaaa@2E64@_3_.

**Table 3:** The effect of measurement error in an exposure on the probability of rejecting the null hypothesis (H_0_) for the test for statistical interaction.

Coefficient for true effect of interaction (*β*_3_)	Ratio for true effect of interaction (*β*_3 _+ *β*_2_)/*β*_2_	Probability of rejecting H_0 _for test of interaction				
						
		*σ*_u_^2 ^= 0	*σ*_u_^2 ^= 1	*σ*_u_^2 ^= 2	*σ*_u_^2 ^= 4	*σ*_u_^2 ^= 9
0.0	1.0	5%	5%	5%	5%	5%
0.5	1.5	87%	54%	38%	26%	16%
1.0	2.0	100%	97%	87%	67%	41%
2.0	3.0	100%	100%	100%	98%	82%

True values of coefficients *β*_1 _= 1 (binary genotype), *β*_2 _= 1 (continuous exposure), *β*_3 _(interaction term) given in first column. No confounding present, *β*_4 _= 0. Simulations based on 10,000 simulations of 1000 observations.

### Practical illustration

The reliability ratios based on the covariance and the measurement error variance matrices estimated from the full regression calibration model were *λ*_x _= 0.82 and *λ*_c _= 0.61 for the exposure and confounder respectively. The correlation between the (imperfectly measured) exposure and confounding variables was 0.15, but the correlation between their predicted true values from the regression calibration was 0.20. Before considering the effect of the confounder (total energy intake), ignoring measurement error in the exposure (haem iron intake) leads to the exposure effect being underestimated by approximately 20% and the interaction with genotype being underestimated by 15%, compared to adjustment for measurement error using regression calibration (Table 4). Adjustment for total energy intake as a confounder, assuming it to be perfectly measured, leads to changes in all the estimates. Expanding the model to allow for confounder measurement error leads to relatively small changes compared to models allowing for exposure measurement error alone. This could indicate, in this simple example with one confounder, that adjusting for an imperfectly measured confounder, ignoring any associated measurement error, may give better estimates than not adjusting for that confounder at all.

**Table 4:** Comparison of methods for handling measurement error in a real dataset using a repeat FFQ on a 33% sub-sample, with total energy intake as potential confounder.

	Without adjustment for total energy intake		With adjustment for total energy intake		With adjustment for total energy intake
	
	Ignoring all measurement error	Regression calibration	Ignoring measurement error	Regression calibration assuming energy intake perfectly measured	Regression calibration allowing for measurement error in energy intake
β^ MathType@MTEF@5@5@+=feaafiart1ev1aaatCvAUfKttLearuWrP9MDH5MBPbIqV92AaeXatLxBI9gBaebbnrfifHhDYfgasaacH8akY=wiFfYdH8Gipec8Eeeu0xXdbba9frFj0=OqFfea0dXdd9vqai=hGuQ8kuc9pgc9s8qqaq=dirpe0xb9q8qiLsFr0=vr0=vr0dc8meaabaqaciaacaGaaeqabaqabeGadaaakeaaiiGacuWFYoGygaqcaaaa@2E64@_0 _**(se)**	3.69 (.02)	3.64 (.02)	4.04 (.05)	4.00 (.05)	4.19 (.08)
β^ MathType@MTEF@5@5@+=feaafiart1ev1aaatCvAUfKttLearuWrP9MDH5MBPbIqV92AaeXatLxBI9gBaebbnrfifHhDYfgasaacH8akY=wiFfYdH8Gipec8Eeeu0xXdbba9frFj0=OqFfea0dXdd9vqai=hGuQ8kuc9pgc9s8qqaq=dirpe0xb9q8qiLsFr0=vr0=vr0dc8meaabaqaciaacaGaaeqabaqabeGadaaakeaaiiGacuWFYoGygaqcaaaa@2E64@_1 _**(se)**	.48 (.20)	.38 (.32)	.42 (.20)	.32 (.32)	.32 (.33)
β^ MathType@MTEF@5@5@+=feaafiart1ev1aaatCvAUfKttLearuWrP9MDH5MBPbIqV92AaeXatLxBI9gBaebbnrfifHhDYfgasaacH8akY=wiFfYdH8Gipec8Eeeu0xXdbba9frFj0=OqFfea0dXdd9vqai=hGuQ8kuc9pgc9s8qqaq=dirpe0xb9q8qiLsFr0=vr0=vr0dc8meaabaqaciaacaGaaeqabaqabeGadaaakeaaiiGacuWFYoGygaqcaaaa@2E64@_2 _**(se)**	.41 (.03)	.51 (.04)	.45 (.03)	.56 (.04)	.57 (.04)
β^ MathType@MTEF@5@5@+=feaafiart1ev1aaatCvAUfKttLearuWrP9MDH5MBPbIqV92AaeXatLxBI9gBaebbnrfifHhDYfgasaacH8akY=wiFfYdH8Gipec8Eeeu0xXdbba9frFj0=OqFfea0dXdd9vqai=hGuQ8kuc9pgc9s8qqaq=dirpe0xb9q8qiLsFr0=vr0=vr0dc8meaabaqaciaacaGaaeqabaqabeGadaaakeaaiiGacuWFYoGygaqcaaaa@2E64@_3 _**(se)**	.88 (.27)	1.04 (.40)	.95 (.27)	1.12 (.39)	1.14 (.39)
β^ MathType@MTEF@5@5@+=feaafiart1ev1aaatCvAUfKttLearuWrP9MDH5MBPbIqV92AaeXatLxBI9gBaebbnrfifHhDYfgasaacH8akY=wiFfYdH8Gipec8Eeeu0xXdbba9frFj0=OqFfea0dXdd9vqai=hGuQ8kuc9pgc9s8qqaq=dirpe0xb9q8qiLsFr0=vr0=vr0dc8meaabaqaciaacaGaaeqabaqabeGadaaakeaaiiGacuWFYoGygaqcaaaa@2E64@_4 _**(se) **(× 1000)	-	-	-.15 (.02)	-.16 (.02)	-.24 (.03)

## Discussion

Under the scenarios considered in this paper, we have shown that measurement error in a confounder can lead to biased estimates of a perfectly measured exposure and that this bias may occur in either direction, dependent on the correlation structure of the data. The mean coefficient estimate of the interaction did not vary with confounder measurement error, but the mean ratio estimate did. This is in contrast to the situation observed for measurement error in an exposure, where the mean coefficient estimate of the interaction varied with exposure measurement error whilst the mean ratio estimate did not. We have confirmed the previously reported lack of effect of exposure measurement error on the ratio estimate of interaction [36], but reveal that the coefficient estimate is biased towards the null in the scenarios considered. Modest amounts of measurement error in the exposure may lead to substantial bias in estimates of the interaction coefficient. The estimated genotype effect is unaffected by measurement error in the confounder.

For the scenarios considered, we also confirm that statistical power to detect the interaction is reduced by measurement error in the exposure [37] and reveal that this is due to attenuated estimates of the interaction coefficient. In addition, we reveal that measurement error in a confounder has no noticeable effect on statistical power for assessing the interaction, whether presented as the coefficient or the ratio estimate. This is because measurement error in the confounder has no noticeable effect on either the estimate of the interaction coefficient or its standard error; it is measurement error only in an exposure (not a confounder) that reduces power to detect an interaction term.

For main effects only, in the absence of interaction, power in detecting an exposure effect is decreased by exposure measurement error in all the scenarios considered. However, confounder measurement error may either increase or decrease the probability of rejecting H_0 _for the main exposure effect, since bias in estimating the exposure effect may occur either towards or away from the null whilst standard errors are virtually unaffected.

The practical illustration demonstrates that relatively large biases may occur due to measurement errors, and this highlights the dangers of ignoring measurement error not only in exposure variables but also in their confounders. However, the strongest impact on the model estimates was adjustment for confounding before taking confounder measurement error into account, illustrating that it is still probably better to adjust for a confounder measured with error than not to adjust for it at all [38]. However, it is important to note that adjustment for covariates which are not true confounders can also lead to bias [39].

Our main result is that random error in a confounder does not influence the estimate of a gene-environment interaction in the situations described, so if the primary goal of a study is estimating the gene-environment interaction, measurement error in a confounder is of lesser importance. However, these results are only directly applicable to situations under the same conditions as the simulations. Other situations are possible:

(i) We have assumed that the genotype is independent of the exposure and confounder, including independence from the exposure variance and exposure error variance. This is not an unreasonable assumption in most epidemiological settings because it is unlikely that genotype will influence an environmental exposure such as dietary intake, or an environmental confounder that is associated with the exposure and the outcome. Similarly, other potential confounders such as age or sex are unlikely to be related to most genotypes under study. However, this assumption must hold for these results to be valid.

(ii) We have also assumed a simple random error model. In nutrition it is quite common for a dietary assessment tool to measure diet with a component of bias and attenuation in addition to random error, such that *W *= *a *+ *bX *+ *U*, where *a *indicates the component of bias in the measured *W *and *b *a component of attenuation multiplying exposure *X*. Whilst regression calibration is able to estimate E(*X|W*) providing an adequate validation measure is available (e.g. a biomarker for the exposure), the combined effects of the different sources of mis-measurement will be more complicated than those described in this paper.

(iii) A further assumption is that there is no genotype by confounder interaction. If this were the case then confounder measurement error would influence the estimate of the genotype by exposure interaction.

(iv) For logistic regression with a binary outcome, the estimated coefficients β^
 MathType@MTEF@5@5@+=feaafiart1ev1aaatCvAUfKttLearuWrP9MDH5MBPbIqV92AaeXatLxBI9gBaebbnrfifHhDYfgasaacH8akY=wiFfYdH8Gipec8Eeeu0xXdbba9frFj0=OqFfea0dXdd9vqai=hGuQ8kuc9pgc9s8qqaq=dirpe0xb9q8qiLsFr0=vr0=vr0dc8meaabaqaciaacaGaaeqabaqabeGadaaakeaaiiGacuWFYoGygaqcaaaa@2E64@_1 _and β^
 MathType@MTEF@5@5@+=feaafiart1ev1aaatCvAUfKttLearuWrP9MDH5MBPbIqV92AaeXatLxBI9gBaebbnrfifHhDYfgasaacH8akY=wiFfYdH8Gipec8Eeeu0xXdbba9frFj0=OqFfea0dXdd9vqai=hGuQ8kuc9pgc9s8qqaq=dirpe0xb9q8qiLsFr0=vr0=vr0dc8meaabaqaciaacaGaaeqabaqabeGadaaakeaaiiGacuWFYoGygaqcaaaa@2E64@_3 _are affected by measurement error in the confounder because of the non-identity link function.

(v) Any measurement error in the genotype will add additional error in the manner of any other exposure, biasing the estimate of the interaction effect.

The suggestion that confounder measurement error has no effect on the estimate of the interaction term under the conditions outlined above does not detract from the impact it may have on other estimates. Confounder measurement error leaves residual confounding that may have a substantial impact on the estimated effect of correlated covariates.

One way to view the effect of confounder measurement error on the estimated interaction effect is to consider the interaction term as allowing the exposure effect to vary across two subgroups defined by genotype (e.g. carriers and non-carriers). The interaction term measures the difference in exposure effect between the two subgroups. Measurement error in a confounder biases the effect of exposure to the same extent in each subgroup, and therefore does not alter the estimated interaction term. If a situation arose in which confounder measurement error differed across the subgroups, perhaps through different data collection procedures, then this would lead to confounder measurement error biasing the estimated genotype by exposure interaction.

Many exposures in nutrition epidemiology have much greater measurement errors associated with them than those in our illustration. Reliability ratios are commonly in the region of 0.3 to 0.5, and even these may underestimate the magnitude of the problem; ratios in the order of 0.1 or 0.2 may be more realistic when derived from models calibrating measured intake against biomarkers [20,40].

## Conclusion

Estimated coefficients for the main effects cannot be assumed to be conservative and only attenuated towards the null in the presence of measurement errors, since errors in confounders may lead to bias in either direction. Measurement error has a more predictable effect on interaction coefficients, which are generally biased towards the null by random measurement error in exposure variables though unaffected by random confounder measurement error in linear regression when genotype can be assumed error-free and independent of exposure and confounder. Despite this, when designing studies where covariates are anticipated to contain measurement error, it is important not only to estimate the measurement error variance of the exposure, but also the measurement error structure of potential confounders. This may have cost implications for large cohort studies where repeated measurements, more labour intensive instruments, or biomarkers may be needed for a large subsample in order to provide adequate precision to adjusted estimates.

## Competing interests

The study of haemochromatosis was funded by the UK Food Standards Agency. The UK Women's Cohort Study was funded by the World Cancer Research Fund. Apart from this, no authors have any competing interests.

## Authors' contributions

DCG had the original idea, designed, conducted and interpreted the simulations and analyses, and wrote the first draft. All authors contributed to further discussion, contributed to subsequent drafts, and approved the final version.

## Pre-publication history

The pre-publication history for this paper can be accessed here:


